# How peptic ulcer disease could potentially lead to the lifelong, debilitating effects of chronic fatigue syndrome: an insight

**DOI:** 10.1038/s41598-021-87018-z

**Published:** 2021-04-06

**Authors:** Chien-Feng Kuo, Leiyu Shi, Cheng-Li Lin, Wei-Cheng Yao, Hsiang-Ting Chen, Chon-Fu Lio, Yu-Ting Tina Wang, Ching-Huang Su, Nai-Wei Hsu, Shin-Yi Tsai

**Affiliations:** 1 Department of Medicine, Graduate Institute of Long-Term Care, Graduate Institute of Biomedical Sciences, Mackay Medical College, New Taipei City, Taiwan; 2 Department of Cosmetic Applications and Management, MacKay Junior College of Medicine, Nursing and Management, New Taipei City, Taiwan; 3 Division of Infectious Diseases, Department of Internal Medicine, Mackay Memorial Hospital, Taipei, Taiwan; 4 Department of Health Policy and Management, Johns Hopkins University Bloomberg School of Public Health, Baltimore, Maryland USA; 5 College of Medicine, China Medical University, Taichung City, Taiwan; 6 Management Office for Health Data, China Medical University Hospital, Taichung City, Taiwan; 7 Department of Anesthesiology and Pain Medicine, Min-Sheng General Hospital, Tao-Yuan, 330 Taiwan; 8 Department of Laboratory Medicine, Mackay Memorial Hospital, Taipei, Taiwan

**Keywords:** Peptic ulcers, Psychology, Risk factors

## Abstract

Chronic Fatigue Syndrome (CFS) has been defined as unexplained relapsing or persistent fatigue for at least 6 consecutive months. Immuno-inflammatory pathway, bacterial infection, and other causes play essential roles in CFS. *Helicobacter pylori* infection is one of the most common causes of foregut inflammation, leading to peptic ulcer disease (PUD). This study aimed to analyze the risk of CFS development between patients with and without PUD. Other related factors were also analyzed. We performed a retrospective, nationwide cohort study identifying patients with or without PUD respectively by analyzing the Longitudinal Health Insurance Database 2000 (LHID2000), Taiwan. The overall incidence of CFS was higher in the PUD cohort than in the non- PUD cohort (HR = 2.01, 95% CI = 1.75–2.30), with the same adjusted HR (aHR) when adjusting for age, sex, and comorbidities. The sex-specific PUD cohort to the non-PUD cohort relative risk of CFS was significant in both genders. The age-specific incidence of CFS showed incidence density increasing with age in both cohorts. There is an increased risk of developing CFS following PUD, especially in females and the aging population. Hopefully, these findings can prevent common infections from progressing to debilitating, chronic conditions such as CFS.

## Introduction

Chronic fatigue syndrome(CFS), also known as Myalgic Encephalomyelitis, was defined by Fukuda et al. in 1994 as patients experiencing relapsing or persistent fatigue due to unexplained etiology for at least 6 consecutive months^1^. Four or more clinical features, such as unusual post-exertion fatigue, unrefreshing sleep, impaired memory or concentration, headache, muscle pain, joint pain, sore throat, and tender cervical nodes should also present as a wide variety of symptoms that extend beyond the major symptom of fatigue^2^. Currently, the accepted practice of diagnosing CFS is based completely on clinical symptoms (often non-specific), without any promising laboratory test or imaging study for confirmation. Thus, diagnosing CFS itself poses a great challenge for general physicians^3^.

To our knowledge, the pathophysiology of CFS still remains unclear, and, with the difficulty in diagnosis, the number of patients involved in the illness are believed to be underestimated^4^. Recent literature had proposed several hypotheses to explain the possible mechanism of CFS, including immuno-inflammatory pathways^5^, neuro-immune dysfunctions^5^, oxidative, and nitrosative stress (O&NS) pathways^6,7^, bacterial translocation^8,9^, gut microbiota dysbiosis^10^, hypothalamic–pituitary–adrenal (HPA) dysfunction, and last but not least, infection of the brain^11^. Genetic-based factors concerning the putative mechanism may also be involved in the interplay between multiple factors of people with CFS^4,12^, which may promote the etiology of CFS to the cellular, molecular level.

Among these factors, the association between CFS and immuno-inflammatory pathways has long captured clinicians’ attention and thus became one of the most intensely studied mechanism of CFS. Surprisingly, two proposed models have been made to clarify the possible etiology and overall course of CFS. The biopsychosocial model reported by Harvey et al. suggested that CFS resulted from a combination of predisposed factors followed by the triggering event, and then a pattern of behavioral and biological responses that lead to CFS^13^. Another possible model demonstrated by Maes, M. et al. expanded on this hypothesis and added the inflammatory, immune, oxidative, and nitrosative (IO&NS) pathways into Harvey’s model. The study further emphasized the importance of viral and bacterial infections, stress from physical and psychosocial, or organic disorders such as immune diseases (especially in the brain, muscles, and the gut), which could also result in CFS^14^. Though both models based on observational studies and the diagnostic criteria of CFS haven’t been fully constructed at that time, these models provide vital clinical findings to inspire further work. In other words, immune-inflammatory factors and postinfectious status can both increase the risk of developing CFS^9,15,16^. Based on the previous findings, we would focus on investigating if peptic ulcer disease (PUD),a prevalent bowel illness mainly due to *Helicobacter pylori* infection worldwide^17^. The *Helicobacter pylori* infection is often acquired early in life and leads to intense cytokine-mediated infiltration of the gastric epithelium by neutrophils and mononuclear cells located in the stomach or proximal duodenum^18^. The gathering and reaction of the inflammatory cells after infection with *Helicobacter pylori* will result in the form of peptic injury, which is termed as the commonly known peptic ulcer disease(PUD)^19^.

In addition, PUD is related to not only physiological symptoms but also functional and mental disorders. A cross-national mental health survey conducted by World Health Organization revealed that prior psychosis experiences were significantly associated with subsequent onset of several general medical conditions including PUD^20^. After PUD develops, fatigue or weakness can be observed among PUD patients especially those sufferings with intermittent abdominal pain or chronic bleeding. Furthermore, PUD may induce sleeping disorders such as less sleeping time or sleep disturbances^21,22^. These clinical presentations are similar to the non-specific clinical manifestations of CFS. Last but not least, chronic and serious *Helicobacter pylori* infections could lead to executive and memory impairment and thus contribute to the neuro-degeneration diseases such as Alzheimer’s disease^23^.

Although the overlapping symptoms and pathophysiological similarities between PUD and CFS are noteworthy, the exact relationships between the two diseases and general population statistics have yet to be established. Thus, the study aims to investigate the association between PUD and new onset CFS. The goal of our population-based retrospective study was to analyze the hazard ratios (HRs) and cumulative incidence rates of CFS between patients with and without PUD by using the National Health Insurance Research Database (NHIRD) of Taiwan. Other related factors including sex, age, and the comorbidities of PUD were also analyzed. To adjust the hazard ratios, we included the comorbidities of CFS, such as diabetes, obesity, renal disease, rheumatoid arthritis, HIV, malignancy, untreated hypothyroidism, sleep apnea, narcolepsy, HBV, HCV, depression, anxiety, bipolar affective disorders, schizophrenia, delusional disorders, dementia, anorexia or bulimia nervosa, and alcohol or other substance abuse. With the significant findings, the prevention of PUD patients suffers from CFS is an important issue in Public Health. We would focus on investigating if peptic ulcer disease (PUD), a prevalent bowel illness due to *Helicobacter pylori* infection and sharing some similar symptoms with CFS including fatigue, sleep disturbance, and memory impairment^21–23^, increases the risk of CFS. We aim to raise the awareness of clinicians to realize the difference between chronic fatigue and CFS^24^ and pay more attention to the risk factors aggravating CFS in patients with PUD in the long run and help them prevent from a lifelong, debilitating condition demanding more resources from the health care systems.

## Materials and methods

### Data source

This retrospective nationwide cohort study analyzed the Longitudinal Health Insurance Database 2000 (LHID2000) of the Taiwan National Health Insurance (NHI) program. This NHI program was implemented in March 1995 and since then has gone on to represent nearly 99% of the 23.74 million Taiwan residents^25^. The details of the NHI program and LHID2000 can be found in previous studies^15,26^. Diagnoses were based on the International Classification of Diseases, 9th Revision, Clinical Modification (ICD-9-CM). The study was approved by the Institutional Review Board of China Medical University and Hospital in Taiwan (CMUH-104-REC2–115) and the Institutional Review Board of MacKay Memories Hospital (16MMHIS074).

### Sample participants

**Figure 1:**
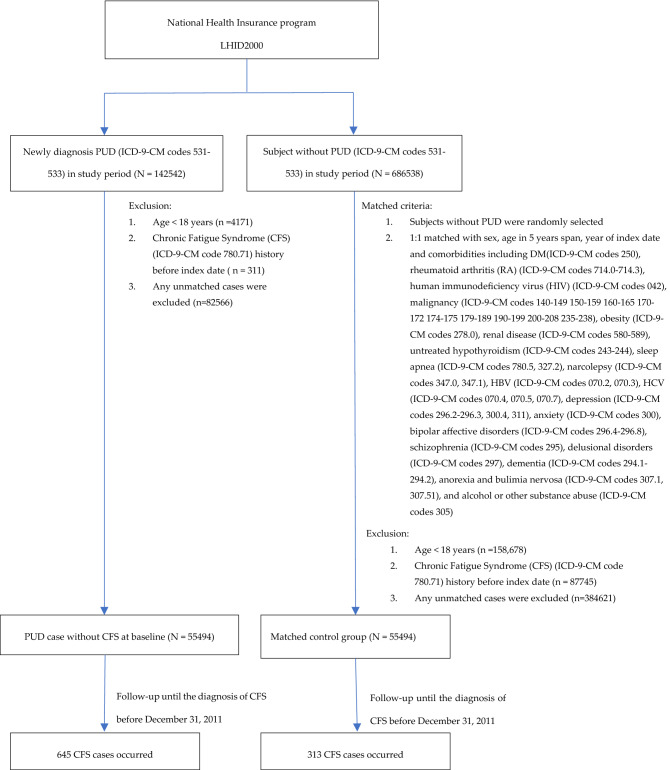
The selection process of PUD-group and non-PUD group. CFS Chronic Fatigue Syndrome, PUD Peptic Ulcer Disease, LHID National Health Insurance Research database, ICD-9-CM International Classification of Diseases, Ninth Revision, Clinical Modification.

Figure 1 is a flowchart indicating how we designed the study. For the PUD cohorts, we identified and included patients aged ≧ 20 years newly diagnosed with peptic ulcer disease (PUD) (ICD-9-CM codes 531–533) with complete age or sex information between 2000 and 2010. The date of diagnosis of PUD was defined as the patient’s index date. To increase the validity of CFS and PUD diagnoses, we selected patients who received outpatient service over twice or inpatient hospitalization at least once. Patients with a history of chronic fatigue syndrome (CFS) (ICD-9-CM code 780.71) before the index date were excluded. One non-PUD control subject with complete age or sex information was frequency-matched with the a PUD cohort on sex, age group (every 5-year span), the year of index date, comorbidities of diabetes (ICD-9-CM codes 250), obesity (ICD-9-CM codes 278.0), renal disease (RD) (ICD-9-CM codes 580–589), rheumatoid arthritis (RA) (ICD-9-CM codes 714.0–714.3), human immunodeficiency virus (HIV) (ICD-9-CM codes 042), malignancy (ICD-9-CM codes 140–149 150–159 160–165 170–172 174–175 179–189 190–199 200–208 235–238), untreated hypothyroidism (ICD-9-CM codes 243–244), sleep apnea (ICD-9-CM codes 780.5, 327.2), narcolepsy (ICD-9-CM codes 347.0, 347.1), HBV (ICD-9-CM codes 070.2, 070.3), HCV (ICD-9-CM codes 070.4, 070.5, 070.7), depression (ICD-9-CM codes 296.2–296.3, 300.4, 311), anxiety (ICD-9-CM codes 300), bipolar affective disorders (ICD-9-CM codes 296.4–296.8), schizophrenia (ICD-9-CM codes 295), delusional disorders (ICD-9-CM codes 297), dementia (ICD-9-CM codes 294.1–294.2), anorexia and bulimia nervosa (ICD-9-CM codes 307.1, 307.51), and alcohol or other substance abuse (ICD-9-CM codes 305) as non-PUD cohort for each PUD case. The same exclusion criteria were applied to the non-PUD cohort. Each study subject was followed until either a diagnosis of CFS was made, patient withdrawal from the NHI program, death, or ultimately until the end of December 31, 2011.

### Statistical analysis

The distributions of sex, age, and comorbidity were compared between the PUD cohort and non-PUD cohort using the Chi-square test for categorical variables and Student’s t-test for continuous variables. We used Kaplan–Meier method to estimate the cumulative incidences of CFS for the PUD cohort and the non-PUD cohort and the log-rank test to compare the difference between the two cohorts. The incidence density rate of CFS for each cohort was estimated to be the number of CFS event occurrence divided by sum of follow-up time (per 1000 person-years). Univariable and multivariable Cox proportional hazards regression analyses were used to assess the CFS risk associated with PUD and estimate the hazard ratios (HRs) with 95% confidence intervals (CIs). The multivariable models included adjusting for age, sex, and comorbidities of diabetes, obesity, renal disease, rheumatoid arthritis, HIV, malignancy, untreated hypothyroidism, sleep apnea, narcolepsy, HBV, HCV, depression, anxiety, bipolar affective disorders, schizophrenia, delusional disorders, dementia, anorexia and bulimia nervosa, and alcohol or other substance abuse. All analyses were performed using the SAS software version 9.4 (SAS Institute Inc., Cary, NC, USA) (URL: https://bityl.co/4Os8), and the significance level was set at 0.05 for the two-tailed tests^15,16,26^.

## Results

### Characteristics of patients

**Figure 2:**
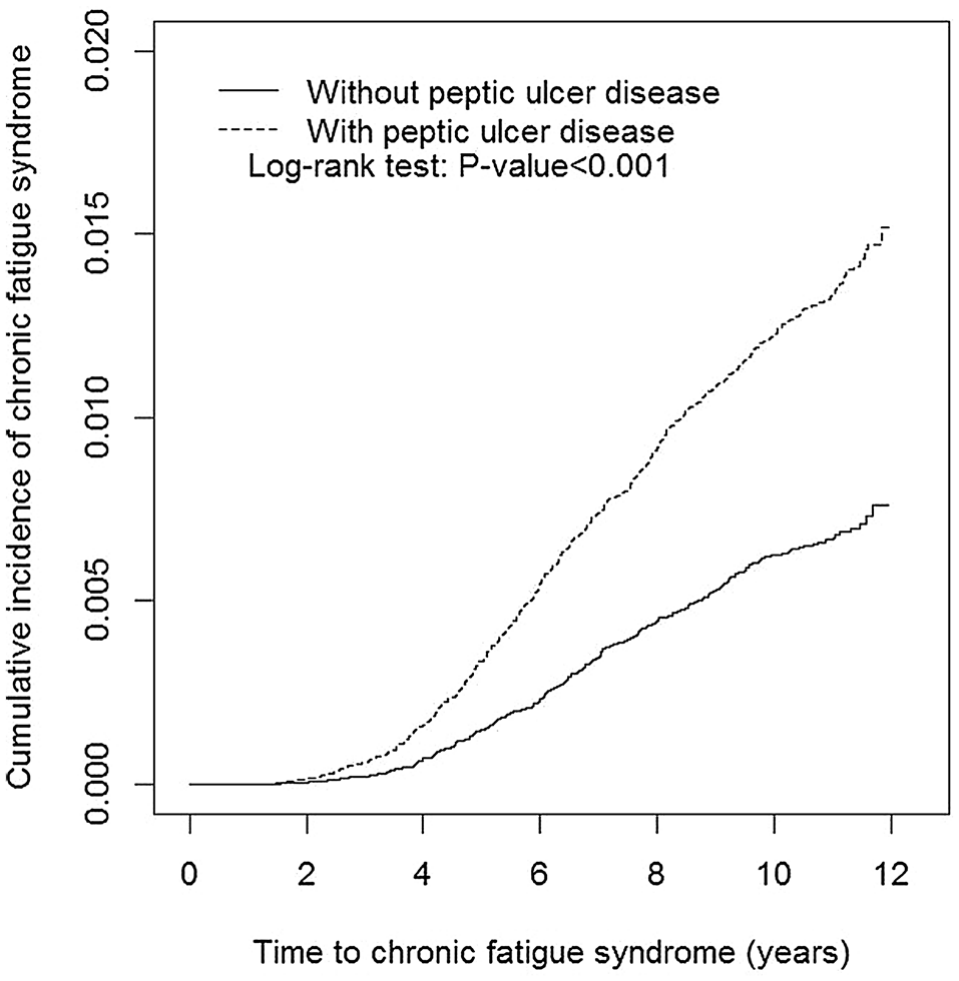
Cumulative incidence of chronic fatigue syndrome compared between patients with and without peptic ulcer disease using the Kaplan–Meier method.

The sex, age, and comorbidity of the PUD cohort (N = 55,494) and non-PUD cohort (N = 55,494) are shown in Table 1. Between the two cohorts, the majority of the patients were female (51.6%) and were aged ≤ 49 years (52.6%). The mean age was 50.1 ± 17.0 years in the PUD cohort and 50.2 ± 17.1 in the non-PUD cohort, respectively. The major comorbidity was anxiety (42.5%) in these study cohorts, followed by diabetes (14.8%), depression (15.2%), renal disease (9.74%), and malignancy (9.16%). The mean follow-up years were 9.60 and 9.40 for the PUD cohort and the non-PUD cohort, respectively (data not shown). Patients with PUD had an approximately 0.75% higher CFS cumulative incidence rate than the non-PUD group after 12 years follow-up (log-rank test p < 0.001, Fig. 2).

**Table 1:** Demographic characteristics and comorbidity in patient with and without peptic ulcer disease.

Variable	Peptic ulcer disease		
	No	Yes	
	N = 55,494	N = 55,494	
**Sex**	n(%)	n(%)	0.99
Female	28,641(51.6)	28,639(51.6)	
Male	26,853(48.4)	26,855(48.4)	
**Age, years**			0.18
20–34	11,487(20.7)	11,672(21.0)	
35–49	17,444(31.4)	17,550(31.6)	
≧50	26,563(47.9)	26,272(47.3)	
Mean(SD)	50.2(17.1)	50.1(17.0)	0.58
**Comorbidity**			
Diabetes	7538(13.6)	8208(14.8)	< 0.001
Obesity	908(1.64)	1273(2.29)	< 0.001
Renal disease	4037(7.27)	5407(9.74)	< 0.001
Rhematoid arthritis	122(0.22)	237(0.43)	< 0.001
HIV	48(0.09)	51(0.09)	0.76
Malignancy	3762(6.78)	5085(9.16)	< 0.001
Untreated hypothyroidism	572(1.03)	1099(1.98)	< 0.001
Sleep apnea	546(0.98)	1257(2.27)	< 0.001
Narcolepsy	27(0.05)	55(0.10)	0.002
HBV	1810(3.26)	4583(8.26)	< 0.001
HCV	764(1.38)	2320(4.18)	< 0.001
Depression	3027(5.45)	8443(15.2)	< 0.001
Anxiety	9726(17.5)	23,558(42.5)	< 0.001
Bipolar affective disorders	208(0.37)	533(0.96)	< 0.001
Schizophrenia	377(0.68)	284(0.51)	< 0.001
Delusional disorders	208(0.37)	277(0.50)	< 0.001
Dementia	2310(4.16)	3191(5.75)	< 0.001
Anorexia or bulimia nervosa	43(0.08)	108(0.19)	< 0.001

Chi-square test; #: Two sample T-test. HIV human Immunodeficiency virus, HBV hepatitis B virus, HCV hepatitis C virus, SD standard deviation.

### Incidence of CFS

The overall incidence of CFS was higher in the PUD cohort than in the non- PUD cohort (1.21 and 0.60 per 1000 person-y, crude HR = 2.01, 95% CI = 1.75–2.30), with an adjusted HR (aHR) of 1.83 (95% CI = 1.59–2.11) when adjusting for age, sex, comorbidities of diabetes, obesity, renal disease, rheumatoid arthritis, HIV, malignancy, untreated hypothyroidism, sleep apnea, narcolepsy, HBV, HCV, depression, anxiety, bipolar affective disorders, schizophrenia, delusional disorders, dementia, anorexia or bulimia nervosa, and alcohol or other substance abuse (Table 2). The sex-specific PUD cohort to the non-PUD cohort relative risk for CFS was significant for both women (aHR = 2.06, 95% CI = 1.69–2.52) and men (aHR = 1.61, 95% CI = 1.31–1.97). The age-specific incidence of CFS showed that the incidence density increased with age in both cohorts; however, the risk for CFS in patients with PUD was higher than non-PUD cohort regardless of age groups. The comorbidity-specific PUD cohort to non-PUD cohort aHR of CFS was significant for patients without comorbidities (aHR = 2.23, 95% CI = 1.80–2.75) or with comorbidities (aHR = 1.71, 95% CI = 1.43–2.05)*.*

**Table 2:** Comparison of incidence and hazard ratio of chronic fatigue syndrome stratified by sex, age and comorbidity between patients with and without peptic ulcer disease.

Variable	Peptic ulcer disease						Crude HR (95% CI)	Adjusted HR† (95% CI)
	No			Yes				
	Event	PY	Rate#	Event	PY	Rate#		
All	313	521,915	0.60	645	532,843	1.21	2.01(1.75, 2.30)***	1.83(1.59, 2.11)***
**Sex**								
Female	158	274,642	0.58	352	282,402	1.25	2.15(1.79, 2.60)***	2.06(1.69, 2.52)***
Male	155	247,273	0.63	293	250,441	1.17	1.86(1.53, 2.26)***	1.61(1.31, 1.97)***
**Stratify age**								
≤ 49	45	111,983	0.40	107	119,112	0.90	2.22(1.56, 3.14)***	2.01(1.39, 2.89)***
50–65	49	175,368	0.52	204	178,893	1.14	2.19(1.71, 2.80)***	2.04(1.57, 2.65)***
65 +	177	234,563	0.75	334	234,839	1.42	1.87(1.56, 2.25)***	1.68(1.38, 2.03)***
**Comorbidity‡**								
No	152	311,769	0.49	196	183,995	1.07	2.18(1.76, 2.69)***	2.23(1.80, 2.75)***
Yes	161	210,146	0.77	449	348,848	1.29	1.66(1.38, 1.98)***	1.71(1.43, 2.05)***

Rate#, incidence rate, per 1,000 person-years; Crude HR, crude hazard ratio.

Adjusted HR†: multivariable analysis including age, sex, and comorbidities of diabetes, obesity, renal disease, rhematoid arthritis, HIV, and malignancy, untreated hypothyroidism, sleep apnea, narcolepsy, HBV, HCV, depression, anxiety, bipolar affective disorders, schizophrenia, delusional disorders, dementia, anorexia or bulimia nervosa, and alcohol or other substance abuse.

Comorbidity‡: Patients with any one of the comorbidities diabetes, obesity, renal disease, rhematoid arthritis, HIV, malignancy, untreated hypothyroidism, sleep apnea, narcolepsy, HBV, HCV, depression, anxiety, bipolar affective disorders, schizophrenia, delusional disorders, dementia, anorexia or bulimia nervosa, and alcohol or other substance abuse were classified as the comorbidity group.

*p < 0.05, **p < 0.01, ***p < 0.001.

### Influence of comorbidities

Compared with the non-PUD patients without these comorbidities, those with only PUD had higher risk of developing CFS (aHR = 2.22, 95% CI = 1.79–2.74), followed by those with only diabetes (less cases), only obesity (aHR = 2.72, 95% CI = 1.11–6.63), and those with only renal disease (aHR = 2.27, 95% CI = 1.33–3.87). Among patients with PUD, those with any two or more of the identified comorbidities were at significantly increased risk of CFS (aHR = 2.52, 95% CI = 2.05–3.10), however exceeding those with any one comorbidity (aHR = 2.34, 95% CI = 1.89–2.89) (Table 3).

**Table 3:** Joint effects for chronic fatigue syndrome between peptic ulcer disease and chronic fatigue syndrome -associated risk factors.

Variable	N	No. of events	Rate#	Adjusted HR†	95% CI
None	32,540	152	0.49	1	(Reference)
Only peptic ulcer disease	18,917	196	1.07	2.22	(1.79, 2.74)***
Only diabetes	0	0	–		
Only obesity	390	5	1.27	2.72	(1.11, 6.63)*
Only renal disease	1310	15	1.29	2.27	(1.33, 3.87)**
Only rheumatoid arthritis	61	0	0		
Only HIV	17	0	0		
Only malignancy	1729	3	0.24	0.45	(0.14, 1.43)
Only untreated hypothyroidism	172	2	1.15	2.27	(0.56, 9.19)
Only sleep apnea	223	0	0		
Only narcolepsy	6	0	0		
Only HBV	840	8	0.94	2.05	(1.01, 4.18)*
Only HCV	251	1	0.39	0.73	(0.10, 5.24)
Only depression	205	3	1.48	2.95	(0.94, 9.26)
Only anxiety	4154	34	0.82	1.53	(1.05, 2.23)*
Only bipolar affective disorders	23	0	0		
Only schizophrenia	154	1	0.71	1.46	(0.21, 10.4)
Only delusional disorders	26	0	0		
Only dementia	689	1	0.2	0.30	(0.04, 2.18)
Only anorexia or bulimia nervosa	18	0	0		
Only alcohol or other substance abuse	91	2	2.19	4.79	(1.01, 1.02)*
PUD with any one comorbidity	17,566	202	1.21	2.34	(1.89, 2.89)***
PUD with any two or more comorbidity	19,011	247	1.36	2.52	(2.05, 3.10)***

Rate# incidence rate.

No., Number; 95% CI 95%, confidence interval; HIV, human immunodeficiency virus; PUD, peptic ulcer disease.

Adjusted HR†: multivariable analysis including age, and sex.

*p < 0.05, **p < 0.01, ***p < 0.001.

## Discussion

Although several diagnostic criteria of CFS based on clinical features have been developed, most failed to identify or inadequately diagnose CFS among the general population^2^. Such a widespread, non-specific clinical condition including physical and mental symptoms are not only detrimental to patients, but also burdens the social economy^27^. A news feature from Nature titled “A reboot for chronic fatigue syndrome research” has reported that although CFS affects about the same number of people with HIV, CFS receives less attention and significantly less funding (about 200 times less than HIV) from the NIH (National Institute of Health) in the United States in 2017^4^. With inadequate medical education on the topic, limited information in medical textbooks, less familiarity for general physicians, diagnosing CFS remains a great challenge for clinicians. More attention should be given to the syndrome in medical school, and further training and workshops should be provided to practicing doctors to develop familiarity in this area^28^.

This study is the first retrospective cohort study to date supporting the association of PUD with a greater risk of developing CFS compared to the non-PUD group, based on a nationwide database in an East- Asian population. In the study group, we found a greater incidence of CFS observed among patients with PUD who were female (51.6%), of older age, and had one comorbidity including diabetes, obesity, renal disease, or any two or more comorbidities (Table 2). Patients with PUD had an approximately 0.75% higher CFS rate than non-PUD after 12 years follow-up (p < 0.001, Fig. 2)*.*

Respectively, our study demonstrated that both genders with PUD were susceptible to develop CFS, with statistical significance. However, we observed that the adjusted HR of women (aHR = 2.06, 95% CI = 1.69–2.52) with PUD developing CFS is higher than in men (aHR = 1.61, 95% CI = 1.31–1.97). Previous studies have focused on gender differences in CFS patients and proposed that differences in incidence rates could be due to gender bias. Several studies showed consistency with our study in that the CFS population is predominantly females^16,29^. Immuno-inflammatory related diseases such as lupus erythematosus and multiple sclerosis among CFS patients showed similar epidemiological gender bias and lower severity of symptoms within the male population when conducted by Mònica et al^30^. Another study by Samantha et al. in Australia focusing on the epidemiological characteristics revealed that patients were predominantly female (78.61%), and the common events such as getting a cold or flu, gastrointestinal illness, periods of undue stress, and infection were present prior to developing CFS symptoms^31^. This is similar with our assessment of gastrointestinal illness such as PUD prior to CFS development.

Our findings revealed that the incidence rate increases with age in both cohorts. In addition, the risk for developing CFS in patients with PUD was higher than the non-PUD cohort, regardless of the age groups. However, we observed a decreasing HR with age in both cohorts. Because we used match analysis, we could not show the effect of age in our study. Since several studies contained a smaller sample size, not many emphasized the increase of incidence density with age, an area which lacks discussion. However, many studies focused on the group of adolescents in which we did not include in our study. Abdominal pain is one of the most common patient presentations to the emergency room in the United States, of which peptic ulcer disease resulting from infection with *Helicobacter pylori* is included^32^. In addition, elderly groups had higher prevalence with PUD and frequently presented with bleeding symptoms^33^. A review article also states that older patients with multiple drug use including antiplatelet medication, selective serotonin reuptake inhibitors, Non-Steroidal Anti-Inflammatory Drugs (NSAIDs), and bisphosphonates are at a high risk of developing PUD^34^. We think the rationale behind the incidence density increase is due to general aging, combined with metabolic disorders accompanied by the poor digestive function resulting from PUD, and higher frequency of medical visits.

The comorbidity-specific PUD cohort to non-PUD cohort aHR of CFS was significant for patients without comorbidity (aHR = 2.23, 95% CI = 1.80–2.75) or with comorbidity (aHR = 1.71, 95% CI = 1.43–2.05). People with metabolic conditions or chronic diseases such as DM and obesity may have strong association with developing CFS, which was consistent with that those with only DM or obesity had high risk of CFS^35^. Moreover, fatigue is recognized as one of the important symptoms of among chronic kidney disease (CKD) or end stage renal disease(ESRD) patients, demonstrated from the findings of that of Manisha et al., who observed up to 84% of prevalence of fatigue among CKD stage 5 patients^36,37^. It also demonstrated that CKD and ESRD patients who experience profound fatigue may be more likely to develop depressive symptoms, restless leg syndrome, excessive daytime sleepiness, and low albumin levels^36^. However, since the comorbidities in the Table 1 involved in PUD or CFS present variously, and few studies focused on their insight mechanisms, so these findings still need more investigations.

Due to the potential of overlapping symptoms with CFS and PUD, this raises the speculation of association between PUD and subsequent CFS. The World Health Organization had recognized CFS as a post-viral fatigue syndrome of the nervous system within ICD-10 since 1992^38,39^. However, there is no convincing explanation that might elaborate the etiology and pathogenesis between CFS and PUD, nor consistent diagnostic criteria. Previously proposed models describe the course of CFS from biopsychosocial to immune-inflammatory aspect, but perhaps this complex disorder may need the aid of translational medicine or genotyping microarrays to explore the molecular aspect^40,41^. Two possible mechanisms are discussed here.

Abnormal adrenocortical activity is one of the mechanisms that link the CFS with PUD. Hypocortisolemia^42^, loss of the diurnal peak of adrenocorticotropic hormone(ACTH) and cortisol levels^43^; blunted responsiveness of HPA axis under the challenge test^44^; recent immune-inflammatory, oxidative, and nitrosative stress (O&NS) pathways^45^ were well reported among CFS. Since 1981, dysregulation of HPA axis was reported among CFS patients^46^, and there were several following studies that were dedicated to the insight between CFS and PUD. The HPA axis releases multiple hormones, especially corticosteroid, that impacts systemic physical, cognitive, emotional, and behavior responses when under stress mentally or physically. Fluctuating corticosteroid level may additionally closely correlate with depression, pain, and fatigue in cancer population^47^. Stress may activate two main vertebrate stress response systems: the HPA axis and the sympatho-medullary system that eventually leads to gastro-duodenal ulceration and inflammatory bowel disease^48^. Furthermore, animal models demonstrated that HPA dysfunction with lower levels of corticosterone, decreased gastric pH, and elevated ACTH levels are seen in peptic ulcer disease^49,50^.

Immuno-inflammatory related factors have become a strongly indicated area of exploring PUD and subsequent CFS. Inflammatory bowel disease (IBD), an intestinal barrier dysfunction, and intestinal flora-induced inflammation in the small intestine, was observed to have a higher incidence in developing CFS^9^. Viruses such as Herpes zoster were found to be a potential group that would be associated with CFS, and anti-viral strategies have been suggested for prevention^16^. Systemic inflammations of skin including atopy and psoriasis were also shown to have an increased risk for subsequent CFS^15,51^. When it comes to immune-inflammatory responses, cytokines play an important role during the whole process.

Based on the mechanisms addressed above, there are some possible implications from a clinical point of view. First, we recommend that physicians should be aware of increased risk of CFS among PUD patients. With the appropriate referrals, the prognosis of patients might improve and the financial cost to health systems might decrease significantly. Secondly, *Helicobacter pylori* infection can alter gastrointestinal microbiome in human bodies, leading to chronic and low degree of inflammatory response^52^. Several microbiome studies have described alterations in the microbiome (dysbiosis) consistent with the mechanism that we discussed to alleviate inflammation and reduce the risk of CFS^53^. According to one animal study, an increase in the protective microbiota, in particular Lactobacillus, might alleviate the advancement of immune deficiency^54^. Physicians can consider probiotic therapies such as *Lactobacillus, Bifidobacterium* and *Saccharomyces* used concomitantly with standard eradication therapy^55^. Thirdly, some plant metabolites, Flavonoids and Coptidis Rhizoma, from gastroprotective Chinese medicinal plants are investigated to have anti-inflammatory effects^56,57^. Through this study, these metabolites may be used as the complementary treatments to cure recurrent and persistent PUD combined with CFS.

However, identifying CFS patients is based on clinical features while diagnostic biomarkers for CFS have not yet been established completely. A systemic review demonstrated the elevation of TGF-β among CFS patients had significant differences between cases and control^58^, while up to thirteen cytokines (including: CCL11, CXCL1, CXCL10, IFN-γ, IL-4, IL-5, IL-7, IL-12, IL-13, IL-17, leptin, G-CSF, and GM-CSF ) were recognized among CFS patients and had a statistically significant upward linear trend that correlated with CFS severity^59^. However, most of these findings are not consistent with each other despite analysis the cytokines level in serum and cerebrospinal fluid within CFS patients^60^. We had previously reported that several systemic immuno-inflammatory diseases such as atopy, psoriasis, varicella-zoster virus reactivation, inflammatory bowel disease, and burn injury could significantly increase the risk for developing CFS in the general population, further highlighting the association between CFS and the disordered immune-inflammatory systems^9,15,16,26,51^. Although the evaluation of immuno-inflammatory factors reflects the hypothesis of the CFS in theory, the etiology is still under debates, and further research may be needed. Thus, although the potential causal effects of PUD on the risk of CFS remain unclear, PUD-induced dysregulation of the gut immune system and chronic systemic inflammation may act as potential mediators that lie in the pathway from PUD to increased risk of CFS. To this end, a statistical approach named Mendelian Randomization has been employed to investigate the potential causal effects of risk factors on risk of certain diseases^61^. Unfortunately, we were unable to link our risk factors to the genetic variant database and therefore were unable to conduct this analysis.

The strength of the study is the large number of patients, about 110,988 people enrolled in total in cases and control groups. The NHI database of Taiwan consists of complete and valid information regarding the demographic characteristics of patients in both groups, thus minimizing the selection bias in our study. We have also considered variables, such as sex, age, comorbidities, and medical treatment and adjust them individually as well. However, our study has some limitations. First, the severity as well as complications of PUD have not been assessed in this study since the severity of PUD needs the images of gastroenterology endoscope. It’s a noteworthy issue needed to use hospital data for further studies.

Second, we did not take medication such as proton-pump inhibitor (PPI), NSAIDs, or histamine 2-receptor antagonists(H2RA) for PUD into consideration due to the anonymity of the data from the NHIRD. Third, our studied population was composed of East Asians in majority living in Taiwan, thus the genetic and geographic discrepancies within the different populations should be examined by further multinational studies. Lastly, we did not discuss the effect of *Helicobacter pylori* eradication, the major treatment for PUD, on the developing risk of CFS. Thus, other researchers can take the effects of PUD treatment into consideration by randomized controlled trial studies.

## Conclusions

This study is the first nationwide population-based study to investigate the risk of CFS in patients with PUD and provides a better understanding of the association between the two conditions. The incidence of CFS was significantly higher in the PUD group than in the non-PUD group, and an increased risk of developing CFS following PUD was observed, especially in females and the aging group. Future studies may aim to assess how severity of PUD and PUD medication, treatments may correlate to CFS. Although our findings cannot contribute to the diagnosis of the CFS, we addressed an important issue concerning public health in that prevention may be essential in preventing PUD from developing into the chronic, debilitating condition of CFS.

## Data Availability

The data underlying this study is from the National Health Insurance Research database (NHIRD). Interested researchers can obtain the data through formal application to the Ministry of Health and Welfare, Taiwan (https://reurl.cc/V6v5yA).

## Abbreviations

ACTH: Adrenocorticotropic hormone
CFS: Chronic fatigue syndrome
CKD: Chronic kidney disease
ESRD: End stage renal disease
HPA: Hypothalamic–pituitary–adrenal
NHIRD: National health insurance research database
NSAIDs: Non-steroidal anti-inflammatory drugs
PUD: Peptic ulcer disease
